# Genomic Landscape and Potential Regulation of RNA Editing in Drug Resistance

**DOI:** 10.1002/advs.202207357

**Published:** 2023-03-13

**Authors:** Xu Zhou, Ramkrishna Mitra, Fei Hou, Shunheng Zhou, Lihong Wang, Wei Jiang

**Affiliations:** ^1^ Department of Biomedical Engineering Nanjing University of Aeronautics and Astronautics Nanjing 211106 P. R. China; ^2^ Department of Pharmacology Physiology, and Cancer Biology Sidney Kimmel Cancer Center Thomas Jefferson University Philadelphia Pennsylvania 19107 USA; ^3^ Department of Pathophysiology School of Medicine Southeast University Nanjing 210009 P. R. China

**Keywords:** drug resistance, microRNA regulations, RNA‐binding proteins, RNA editing, therapeutic targets

## Abstract

Adenosine‐to‐inosine RNA editing critically affects the response of cancer therapies. However, comprehensive identification of drug resistance‐related RNA editing events and systematic understanding of how RNA editing mediates anticancer drug resistance remain unclear. Here, 7157 differential editing sites (DESs) are identified from 98 127 informative RNA editing sites in tumor tissues, many of which are validated in cancer cell lines. Diverse editing patterns of DESs are discovered in resistant samples, which could not be fully explained by adenosine deaminase acting on RNA enzymes. Some RNA‐binding proteins are identified that potentially regulate these editing events. Notably, the DESs are significantly enriched in 3’‐untranslated regions (3’‐UTRs). The impact of DESs in 3’‐UTR on the microRNA (miRNA) regulations is explored, and some triplets (DES, miRNA, and gene) that may contribute to drug resistance are identified. In addition, it is determined that the functions of genes enriched with DESs are associated with drug resistance, such as apoptosis, drug metabolism, and DNA synthesis involved in DNA repair. An online resource (http://www.jianglab.cn/REDR/) to support convenient retrieval of DESs is also built. The findings reveal the landscape and potential regulatory mechanism of RNA editing in drug resistance, providing new therapeutic targets for reversing drug resistance.

## Graphical abstract

The study identifies RNA editing events related to drug resistance through a systematic analysis across 6 types of cancer and 18 drugs. The study highlights that RNA editing is a crucial mediator in the mechanism of anticancer drug resistance, substantially improving the understanding of drug resistance mechanisms regarding transcriptional and post‐transcriptional regulation.

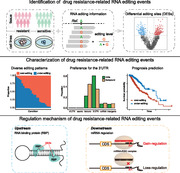

## 1 Introduction

Currently, chemotherapy and molecularly targeted therapies are the primary treatment options to improve the overall survival of patients with cancer.^[^ ^1^ ^]^ However, therapeutic resistance remains a major problem in cancer treatment and a principal cause of poor prognosis across various cancers.^[^ ^2^ ^]^ Recent studies have indicated that the mechanisms of drug resistance in cancer are multifaceted, such as alterations of drug metabolism^[^ ^3^ ^]^ and drug targets,^[^ ^4^ ^]^ DNA damage repair,^[^ ^5^ ^]^ abnormal tumor microenvironment,^[^ ^6^ ^]^ dysregulated noncoding RNAs,^[^ ^7^ ^]^ genetic heterogeneity,^[^ ^8^ ^]^ epigenetic changes,^[^ ^9^ ^]^ and RNA modifications.^[^ ^10^ ^]^

The post‐transcriptional modification of RNA is an important process affecting cellular function as it alters the transcriptional landscape.^[^ ^11^ ^]^ In mammals, adenosine‐to‐inosine (A‐to‐I) RNA editing, a widespread post‐transcriptional modification, is catalyzed by adenosine deaminase acting on RNA enzymes (ADARs) that diversifies the transcriptome by altering selected nucleotides in RNA molecules.^[^ ^12^ ^]^ With the rapid development of high‐throughput sequencing technologies, ≈4.5 million A‐to‐I RNA editing sites have currently been identified.^[^ ^13^ ^]^ Some studies have revealed that altered editing events have serious effects on human health, such as immune disorders,^[^ ^14^ ^]^ neurological disorders,^[^ ^15^ ^]^ cardiovascular disease,^[^ ^16^ ^]^ and cancer progression.^[^ ^17^ ^]^ Additional editome‐disease associations have been shown in the Editome Disease Knowledgebase.^[^ ^18^ ^]^ Collectively, A‐to‐I RNA editing plays critical roles in several biological processes and human diseases.

Recently, the roles of RNA editing have been studied for genes associated with pharmacokinetics and pharmacodynamics.^[^ ^19^ ^]^ Some studies provide evidence that specific RNA editing events could selectively affect the response of cancer therapies, suggesting RNA editing as a predictive marker for therapy response. For example, RNA editing events in the coding region of *COG3* and *GRIA2* increase sensitivity to MEK inhibitors.^[^ ^20^ ^]^ In addition, upregulation of DHFR expression could enhance cellular proliferation and resistance to methotrexate. RNA editing destroys the binding sites of miR‐25‐3p and miR‐125a‐3p in the 3’‐untranslated region (3’‐UTR) of *DHFR*, resulting in elevated *DHFR* levels.^[^ ^21^ ^]^ In some cases, A‐to‐I editing could cause aberrant splicing of targeted mRNA resulting in drug resistance.^[^ ^22^ ^]^ Collectively, A‐to‐I RNA editing has the potential to become a new nucleic acid‐based therapeutic target to reverse drug resistance in cancer. However, because of the limited number of examined RNA editing sites, drugs, samples, or tissues, published studies did not thoroughly analyze RNA editing‐mediated mechanisms in drug response. Thus, systematic analysis of A‐to‐I editing associated with drug response is urgent and necessary.

Here, we aimed to identify RNA editing events related to drug resistance through a systematic analysis across six types of cancer and 18 drugs. There is no significant difference in the overall RNA editing levels (OELs) between resistant and sensitive samples, which was consistent in cancer cell lines. Furthermore, we identified differential editing sites (DESs) in resistant samples relative to sensitive samples. We observed that these DESs were significantly enriched in 3’‐UTRs, whereas a very small fraction were in coding regions. Altered RNA editing patterns (over‐editing or under‐editing) were evidently diverse across conditions (one drug in a specific type of cancer was defined as a condition), which could not be fully explained by ADARs. However, these DESs could share common sequence motifs and were located in binding sites of RNA‐binding proteins (RBPs) related to drug resistance, which indicated that RBPs as upstream regulators may influence the editing activity of these DESs. We also identified RNA editing‐dependent microRNA (miRNA) regulations potentially affecting drug response. In addition, we found that some genes significantly enriched with DESs were associated with drug resistance, such as *TEP1*. Functional analysis of genes enriched with DESs revealed strong functional specificity in different conditions. Finally, we constructed an omnibus repository named REDR, which furnishes a user‐friendly interface for a convenient retrieval of our findings (freely available at http://www.jianglab.cn/REDR/). Our findings highlight that RNA editing is a crucial mediator in the mechanism of anticancer drug resistance, substantially refining our understanding of the RNA editing‐mediated mechanism involved in drug resistance.

## 2 Results

### 2.1 Overall RNA Editing Levels between Resistant and Sensitive Tumor Tissues

**Figure 1:**
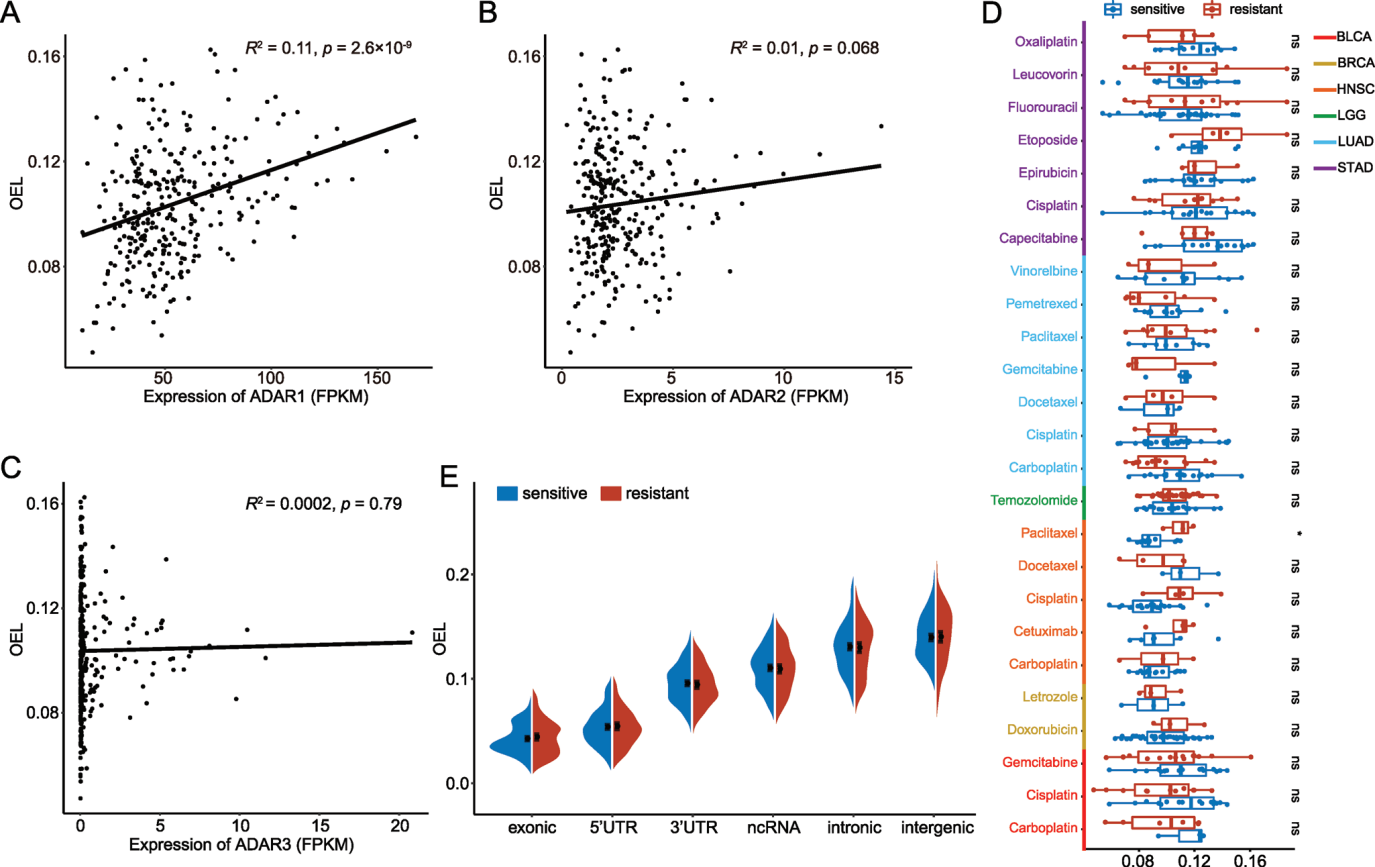
The overall RNA editing levels (OELs) between resistant and sensitive tumor samples. Correlations between expression levels (fragments per kilobase of transcript per million fragments mapped, FPKM) of A) ADAR1, B) ADAR2, or C) ADAR3 and OELs separately across all available tumor samples. *R* ^2^ values were calculated using linear regressions, and *p* represents statistical significance. D) Wilcoxon rank‐sum test was used to test for differences of the OELs between compared groups. Particularly, “*” represents *p* < 0.05, “**” represents *p* < 0.01, “***” represents *p* < 0.001, and “ns” represents “not significant”. E) The comparison of OELs in different transcribed regions between resistant and sensitive samples.

To explore the associations between RNA editing and drug response, RNA editing data and drug response data of tumor tissues of patients across various types of cancer were first downloaded from Synapse and the Genomic Data Commons (GDC) Data Portal, respectively. Through filtering (see Section 4), we obtained 25 conditions across six types of cancer and 18 anticancer drugs (Figure S1, Supporting Information). Thereafter, the OEL was calculated for each sample, defined as the average of the editing levels over all informative RNA editing sites. We discovered that ≈11% of the variation in OELs was explained by ADAR1 (*p* = 2.6 × 10^−9^, **Figure** **1**A; see Section 4). The expression of ADAR2 also explained ≈1% of the variation in OELs (*p* = 6.8 × 10^−2^, Figure 1B). However, the expression of ADAR3 had no significant effect on OELs (*p* = 0.79, Figure 1C), consistent with the previous finding.^[^ ^23^ ^]^ These results suggested that the data are qualitatively good for downstream analysis. We observed that in 96% of the conditions, OELs were not significantly different between resistant and sensitive samples (Figure 1D). Additionally, we calculated the OELs for each transcribed region. We found that the OELs were highest in the intergenic regions and lowest in exonic regions (Figure 1E), and an independent study on brain tissue supported the results.^[^ ^24^ ^]^ Difference in OELs was not evident for distinct transcribed regions among the compared groups (Figure 1E).

Similar to tumor tissue, OEL analysis was performed on cell line data. Based on the drug response data of cancer cell lines from Genomics of Drug Sensitivity in Cancer (GDSC), 14 conditions, same as tumor tissues, were obtained. Each condition includes at least three cell lines in the resistant and sensitive groups (see Section 4; Figure S2A, Supporting Information). Thereafter, RNA‐sequencing (RNA‐seq) data and drug response data were retrieved from the Cancer Cell Line Encyclopedia (CCLE) and GDSC separately. We detected the RNA editome for 102 cell lines from 5 types of cancer by utilizing REDItools (see Section 4). Here, a collection of high‐confidence RNA editing events were identified in cell lines, and a large fraction (86–99%) were also detected in tumor tissues of patients (Table S1, Supporting Information). The cell line data showed similar results concerning the OEL as investigated in tumor tissues (Figure S2B–F, Supporting Information). Collectively, there is no significant difference in the OEL between sensitive and resistant samples for both tumor tissues and cell lines.

### 2.2 Identification and Characterization of Differential Editing Sites

#### 2.2.1 Identification of Differential Editing Sites

**Figure 2:**
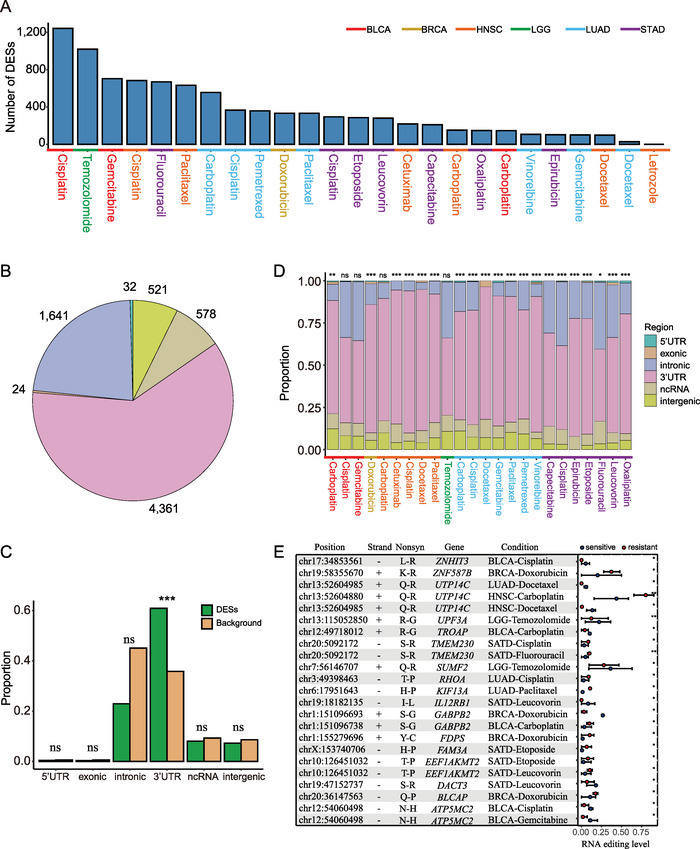
Transcribed region distribution of differential editing sites (DESs). A) The number of DESs under each condition. B) The pie chart shows the distribution of integrative DESs in the transcribed region under all conditions. DESs significantly enriched in the 3’‐UTR rather than other transcribed regions in an C) integrative manner and D) each condition. The *p*‐values were computed using the hypergeometric test. E) The alterations of amino acids were attributed to non‐synonymous DESs in coding regions. The Wilcoxon rank‐sum test was used to detect RNA editing sites with differential editing levels between resistant and sensitive tumor samples. Particularly, “*” represents *p* < 0.05, “**” represents *p* < 0.01, “***” represents *p* < 0.001, and “ns” represents “not significant”.

The global A‐to‐I RNA editing differences between resistant and sensitive tumor tissues remain largely uncharacterized. To comprehensively detect RNA editing events related to drug response, we identified RNA editing sites with significantly differential editing activity between resistant and sensitive tumor tissues under each condition (Wilcoxon rank‐sum test *p* < 0.05 and mean editing‐level difference |*Diff*| ≥ 5%, Table S2, Supporting Information). The number of DESs identified in each condition varied from 0 to 1242 (**Figure** **2**A). In particular, no DESs were identified in breast invasive carcinoma (BRCA)‐Letrozole, which may be because of the use of only a few samples (*n* = 6). Thereafter, we examined whether DESs related to drug response in tumor tissues could be reproduced in cancer cell lines. As expected, DESs identified in cell lines were significantly enriched with DESs from tumor tissues (Table S3, Supporting Information, hypergeometric test, 11/14 conditions with *p* < 0.05). We determined that a considerable number of overlapped genes harboring DESs between the identical condition in cell lines and tumor tissues (Table S4, Supporting Information, 28–82% overlaps). The results indicated that the identified DESs associated with drug response were cross‐validated across tumor tissues and corresponding cancer cell lines. In particular, we observed that the DESs on *DHFR* were located in the 3’‐UTR (Table S2, Supporting Information). A previous study has demonstrated that dysregulation of RNA editing sites in the 3’‐UTR of *DHFR* influences the binding of miR‐25‐3p and miR‐125a‐3p, enhancing cellular proliferation and resistance to methotrexate in MCF‐7 cells.^[^ ^21^ ^]^ We also found that the DESs on *COG3* are located in the coding region. Liang et al. confirmed that RNA editing in the coding region of *COG3* alters drug sensitivity using a high‐throughput Ba/F3 differential cytotoxicity screen.^[^ ^20^ ^]^ These evidence directly support our results.

#### 2.2.2 Differential Editing Sites Prefer the 3’‐Untranslated Region

Thereafter, we next examined the distribution of DESs in the transcribed region in an integrative manner and under each condition separately. Most DESs were found in the 3’‐UTR and intronic regions (Figure 2B). Furthermore, the DESs were predominantly enriched in the 3’‐UTR rather than other transcribed regions with statistical significance in an integrative manner (hypergeometric test, *p* < 2.2 × 10^−16^, Figure 2C; Table S5, Supporting Information) and under each condition (Figure 2D; Table S5, Supporting Information). Nakano et al. have demonstrated that the alteration of RNA editing in the 3’‐UTR of a particular gene could impact the response of anticancer drugs.^[^ ^21^ ^]^ These findings were also noticed under conditions involving cell lines, although the largest proportion of DESs was in the intronic region (Figure S3, Supporting Information). Overall, these results may indicate that RNA editing possibly determines drug response via post‐transcriptional modulations.

By contrast, the number of DESs in the coding region was relatively limited for both tumor samples (≈0.3%) and cell lines (≈0.1%). There were only 19 unique nonsynonymous DESs and five synonymous DESs across 14 conditions from tumor tissue data. Previous studies have reported that A‐to‐I RNA editing events in transcripts of drug targets, metabolizing enzymes, transporters, and transcription factors may affect the drug response.^[^ ^19a^ ^]^ Hence, we displayed amino acid substitutions attributed to the total nonsynonymous DESs under each condition from tumor tissue data (Figure 2E). Moreover, most (76.5%) of these genes were confirmed to be associated with cancer progression and drug resistance (Table S6, Supporting Information). For example, we determined a higher editing level in the coding region of *RHOA* in resistant samples relative to sensitive samples in the Cisplatin‐treated lung adenocarcinoma (LUAD) samples (LUAD‐Cisplatin). A previous study has demonstrated that downregulation of *RHOA* alone results in Cisplatin resistance.^[^ ^25^ ^]^

#### 2.2.3 Prognosis‐Related Differential Editing Sites

**Figure 3:**
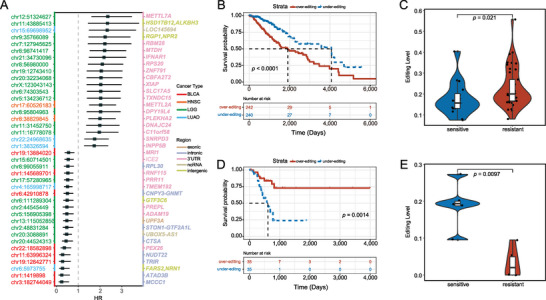
Associations between differential editing sites (DESs) and overall survival of patients. A) Forest plot shows the HRs of top significant prognosis‐related DESs and their corresponding gene information. The over‐editing of two sites was detected as a risk and protective factor separately, consistent with their editing patterns in resistant samples. B) Plot of Kaplan–Meier curves. Patients with LGG that suffered from the higher editing levels on chr12:51324627 show poor prognoses. The *p*‐value was calculated using the log‐rank test. C) Patients with LGG resistant to Temozolomide showed significantly higher editing levels on chr12:51324627 (*p* = 0.021), and the Wilcoxon rank‐sum test was used to calculate the *p*‐value. D) Patients with BLCA with the lower editing level on chr1:1419898 show poor prognoses. E) Patients with BLCA resistant to Cisplatin show significantly lower editing levels on chr1:1419898 (*p* = 0.0097).

Generally, patients with therapy resistance have poor survival outcomes. To measure the associations between the DESs and prognosis, we identified prognosis‐related RNA editing events using the univariate Cox proportional hazards regression model. We found that 859 of 7157 unique DESs had significant (log‐rank test, *p* < 0.05; Table S7, Supporting Information) association with the overall survival in the studied patient cohorts from The Cancer Genome Atlas (TCGA). The corresponding hazard ratios (HRs) of these top prognosis‐linked DESs and associated genes are shown in **Figure** **3**A. Notably, most (82.77%) of these prognosis‐linked DESs have consistent editing patterns in patients with poor prognosis and therapy resistance (Table S7, Supporting Information). For example, higher‐editing levels of chr12:51324627, located in the 3’‐UTR of *METTL7A*, correlate with poor overall survival of patients with lower grade glioma (LGG) (Figure 3B). The same chromosomal position had significantly (*p* = 0.021, Wilcoxon rank‐sum test) higher editing levels in patients with LGG resistance to Temozolomide than those that were LGG sensitive (Figure 3C). Reportedly, increased expression of *METTL7A* was observed in methotrexate‐resistant cancer cell lines and choriocarcinoma tissues.^[^ ^26^ ^]^ In contrast, the under‐editing of chr1:1419898 in the intronic region of *ATAD3B* was a risk factor for patient survival in bladder urothelial carcinoma (BLCA) (Figure 3D). Notably, patients with BLCA resistant to Cisplatin also showed significantly (*p* = 0.0097, Wilcoxon rank‐sum test) lower editing levels on chr1:1419898 (Figure 3E). *ATAD3B* is a human embryonic stem cell‐specific mitochondrial protein, which has been used as the prognostic biomarker for hepatocellular carcinoma^[^ ^27^ ^]^ and BLCA.^[^ ^28^ ^]^ Collectively, these findings suggested that RNA editing may be an important determinant of mediating anticancer drug response.

#### 2.2.4 Editing Patterns of Differential Editing Sites

Thereafter, we compared DESs under different conditions by calculating the Simpson's index (as Meet/Min score) between any two conditions (Figure S4A, Supporting Information, Section 4). We observed that most DESs (≈78%) only exist in a condition, indicating that most DESs were condition‐specific. Unexpectedly, there were a significant number of overlapped genes harboring DESs between any two conditions, which implied that the determinant of drug response might be a complex regulatory process that involved multiple genes harboring DESs (Figure S4B, Supporting Information). Figure S4C, Supporting Information, showed which Gene Ontology (GO) terms overlapped between any two conditions. In addition, markedly more intersected genes and GO terms were observed between two conditions of the same type of cancer (Figure S4B,C, Supporting Information), which was also evident in cell lines (Figure S4D–F, Supporting Information).

**Figure 4:**
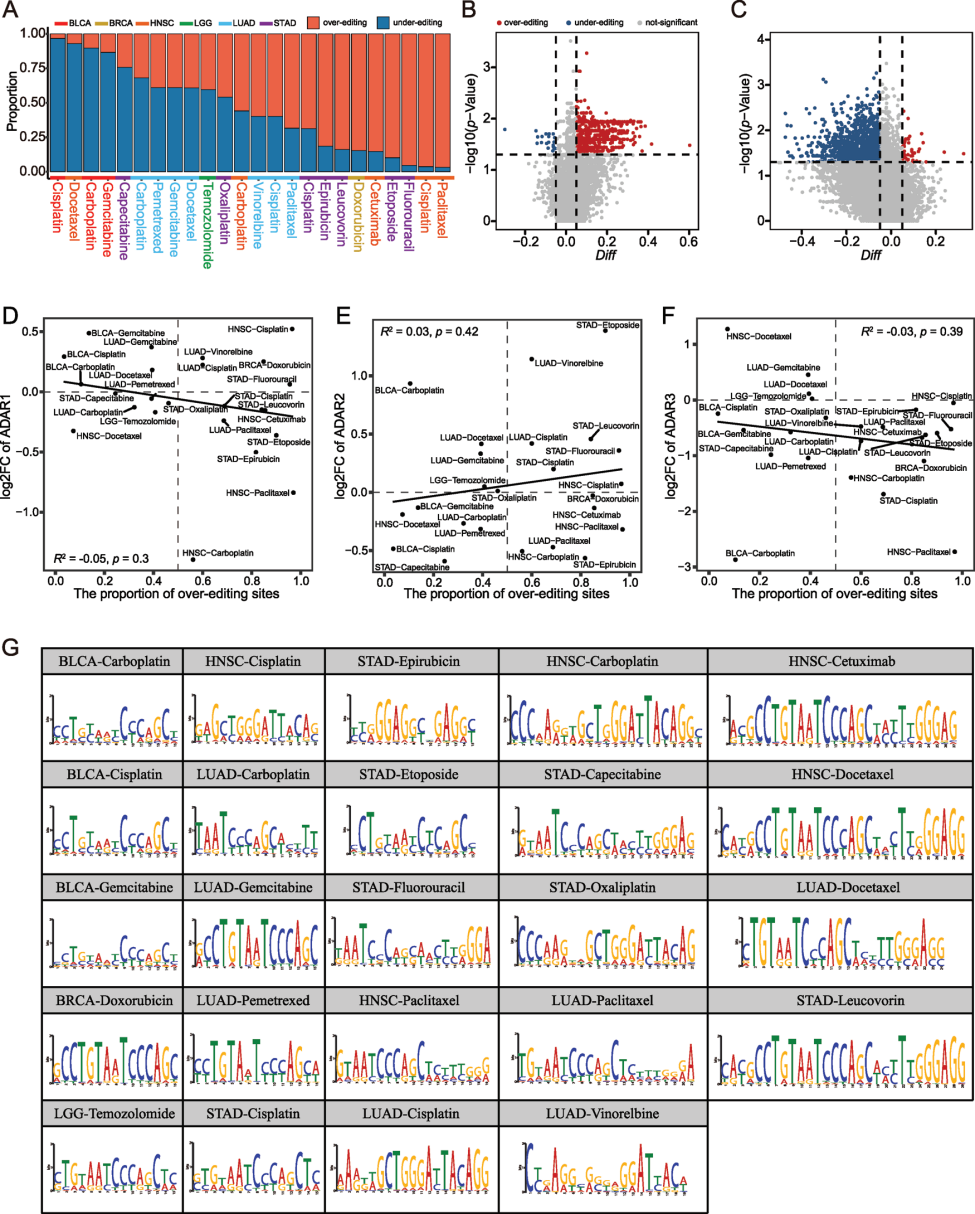
Identification of RNA‐binding proteins (RBPs) that potentially regulate the editing levels of differential editing sites (DESs). Editing patterns of DESs. A) The proportions of over‐editing and under‐editing sites in resistant samples were diverse under all conditions. The volcano plots of differential editing analysis using the Wilcoxon rank‐sum test in B) HNSC‐Paclitaxel and C) BLCA‐Cisplatin. The Pearson correlation was calculated between the proportion of over‐editing sites and log2FC (log2 fold change) of D) ADAR1, E) ADAR2, or F) ADAR3 expression in resistant samples relative to sensitive samples. *R* ^2^ values were calculated using linear regressions, and *p* represents statistical significance. G) The most statistically significant motif was identified under each condition using multiple Em motif elicitation (MEME) analysis.

Subsequently, we counted the over‐ and under‐editing proportions of DESs under each condition. We observed diverse RNA editing patterns for these DESs across conditions. For example, 613 (≈97%) DESs showed significant (*p* < 0.05) over‐editing in resistant tumor tissues compared to sensitive tumor tissues in head and neck squamous cell carcinoma (HNSC)‐Paclitaxel. In contrast, only 20 (≈3%) DESs showed significant under‐editing in resistant samples (**Figure** **4**A,B). Unlike HNSC‐Paclitaxel, in BLCA‐Cisplatin, only 42 (≈3%) DESs showed significant over‐editing in resistant samples, whereas 1200 (≈97%) showed significant under‐editing in resistant samples (Figure 4A,C).

### 2.3 Identification of RNA‐Binding Proteins that Potentially Regulate the Editing Level of Differential Editing Sites

To identify potential regulators underlying editing patterns, we first explored the associations between ADAR enzymes and the observed patterns. We measured correlations between the proportion of over‐editing DESs and the difference of ADAR expression (log2FC) in resistant samples relative to sensitive samples. The poor correlation scores indicated that ADAR enzyme levels could not fully explain these altered RNA editing patterns in resistant samples across conditions (Figure 4D–F). Thus, these results implied that there were possibly some other potential mediators affecting drug response by regulating RNA editing. Notably, recent studies have reported that RBPs can impact editing levels by competing for substrates with ADARs, prompting us to identify potential regulatory factors that account for the editing patterns in resistant samples. Hence, we examined whether DESs shared a common sequence motif for editome recognition. Multiple Em motif elicitation (MEME) analysis identified the most statistically significant motif in the region adjacent to the DESs (a sequence with ±20 bp centered on the DESs) under each condition (Table S8, Supporting Information, Figure 4G, Section 4). Therefore, we observed a consistent 13 nucleotide motif (CCTGTAATCCAGC) under most conditions. Notably, using the tool FIMO,^[^ ^29^ ^]^ our analysis revealed that this motif overlapped with many Alu repetitive elements. The results are consistent with previous findings that Alu repetitive elements are the most frequent and widespread targets of A‐to‐I RNA editing.^[^ ^13a^ ^]^

Subsequently, we also explored whether DESs located in these sequence motifs could be bound by any known human RBPs under each condition. A total of 132 RBPs were identified under all conditions (Table S9, Supporting Information), and RBPs overlap between any two conditions was from 45% to 100%. This indicated that the identified RBPs were not condition‐specific (Figure S5A, Supporting Information). Furthermore, through functional enrichment analysis, we discovered that these RBPs participated in the functions associated with drug resistance, such as mRNA splicing, translation, localization, metabolic process, etc. (Figure S5B, Supporting Information).^[^ ^30^ ^]^ Notably, we found that some RBPs have been confirmed experimentally to regulate RNA editing sites. For example, overexpression of ILF2 reduced editing levels of 11 sites in HEK293T cells.^[^ ^31^ ^]^ TARDBP knockdown induced a global reduction in RNA editing levels in HepG2 cells.^[^ ^32^ ^]^ Moreover, some identified RBPs had been confirmed to be associated with anticancer drug resistance. For example, *FXR1*, encoding an RNA‐binding protein that interacts with functionally‐similar proteins, was identified under the Cisplatin‐related conditions in this study. A previous study reported that the FXR1/PRKCI complex could activate the extracellular signal‐related kinase signaling to promote cell growth and invasion in Cisplatin‐resistant non‐small‐cell lung carcinoma cells.^[^ ^33^ ^]^ Collectively, these results suggested that RBP, as an upstream regulator, may mediate drug response by regulating RNA editing.

### 2.4 Analysis of MicroRNA Regulations Mediated by Differential Editing Sites

Previous research revealed the impact of miRNA‐mediated regulation on drug targets and metabolism‐related genes, which indicated that the post‐transcriptional regulation mechanism is an important determinant of drug efficacy and toxicity.^[^ ^19a^ ^]^ Moreover, altered RNA editing in the 3’‐UTR of genes has the potential to change the expression of the gene via miRNA‐mediated regulation.^[^ ^21^ ^]^ In this study, we discovered that DESs were significantly enriched in the 3’‐UTR (Figure 2B–D and Figure S3, Supporting Information). In particular, we observed a moderate correlation between the RNA editing level of sites and the expression of genes harboring these sites (Figure S6, Supporting Information). Additionally, under some conditions, genes harboring DESs in their 3’‐UTR were significantly enriched with differentially expressed genes (DEGs) between resistant and sensitive samples (Figure S7, Supporting Information). These results suggested that RNA editing possibly regulated gene expression via miRNA modulation mechanisms.

**Figure 5:**
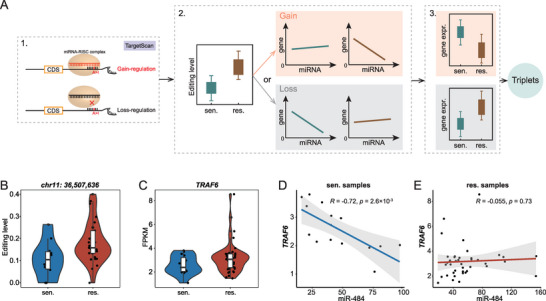
Analysis of miRNA regulations mediated by drug response‐associated RNA editing. A) The diagrams of the multi‐step screening process identifying triplets (DES, miRNA, and gene). B–E) The triplet, chr11:36507636_miR‐484_*TRAF6*. B,C) Comparison of the editing levels on chr11:36507636 and the expression of *TRAF6* between resistant and sensitive samples separately. D,E) The Pearson correlation between miR‐484 and *TRAF6* expression in resistant and sensitive samples, respectively. Here, “res.” represents “resistant,” and “sen.” represents “sensitive.”

Subsequently, through a multi‐step screening process (**Figure** **5**A, see Section 4), we identified RNA editing‐dependent post‐transcriptional regulation triplets (DESs, miRNAs, and genes) across different conditions (Table S10, Supporting Information). For example, in the triplet (chr11:36507636_miR‐484_*TRAF6*) predicted in LGG‐Temozolomide, we observed the over‐editing of chr11:36507636 falling within the miR‐484 binding region in the 3’‐UTR of *TRAF6*, and the over‐expression of *TRAF6* in resistant samples (Figure 5B,C). In addition, we found a significantly negative expression correlation in sensitive samples and no significant correlation in resistant samples between miR‐484 and *TRAF6* (Figure 5D,E). It indicated that the over‐expression of *TRAF6* was possibly attributed to a lack of miR‐484_*TRAF6* regulation due to the over‐editing of chr11:36507636. A previous study uncovered that over‐expression of *TRAF6* could enhance the glioblastoma cell resistance against Temozolomide.^[^ ^34^ ^]^ These results revealed the potential pharmacological consequences of perturbing miRNA regulations depending on A‐to‐I RNA editing. In summary, our results analyzed the driving force of gene expression regulation related to therapy resistance from the perspective of RNA editing.

### 2.5 Biological Insights and Editing Patterns of Genes Enriched with Differential Editing Sites

To analyze the functions in which the DESs might be involved, we first focused on the genes harboring DESs under each condition. Previous findings revealed that the length of genes directly correlates with the total number of detected RNA editing sites on these genes.^[^ ^24^ ^]^ Thus, we examined whether any genes were significantly enriched with DESs beyond what could be expected by chance. Here, we calculated the over‐representation of DESs within each gene by setting a changing background specific to the total number of informative RNA editing events for a particular gene. Therefore, the genes significantly enriched with DESs were identified under each condition using the hypergeometric test (*p* < 0.05, Table S11, Supporting Information). We further discovered that some of these genes significantly enriched with DESs were associated with drug resistance, such as *TEP1*,^[^ ^35^ ^]^ *UTP14C*,^[^ ^36^ ^]^ and *RASAL2*.^[^ ^37^ ^]^

Subsequently, functional enrichment analysis was performed on the genes significantly enriched with DESs under each condition. We observed that some significantly enriched functions and pathways were associated with drug resistance (*p* < 0.05, Tables S12 and S13, Supporting Information), such as the hyperosmotic response,^[^ ^38^ ^]^ mRNA processing,^[^ ^39^ ^]^ apoptosis,^[^ ^40^ ^]^ drug metabolism,^[^ ^41^ ^]^ Wnt signaling pathway,^[^ ^42^ ^]^ chemokine signaling pathway,^[^ ^43^ ^]^ DNA synthesis involved in DNA repair,^[^ ^44^ ^]^ etc. Experiments confirmed that the overall DNA repair capacity of resistant non‐small cell lung cancer cells is significantly elevated.^[^ ^44^ ^]^ Furthermore, some genes significantly enriched with DESs in the above‐mentioned pathways have been experimentally confirmed to be related to drug resistance. For example, the X‐linked inhibitor of apoptosis protein (XIAP) is known to modulate apoptosis by inhibiting caspases and ubiquitinating target proteins. Transfection with XIAP variants conferred resistance to doxorubicin and increased cellular proliferative capacity in MCF‐7 cells.^[^ ^45^ ^]^ Additionally, EGFR tyrosine kinase inhibitor resistance and endocrine resistance pathways were significantly enriched, implying that RNA editing possibly mediates drug resistance for molecular targeted and endocrine therapies. Furthermore, we observed that the significantly enriched GO terms and Kyoto Encyclopedia of Genes and Genomes (KEGG) pathways were condition‐specific (Figure S8A,B, Supporting Information). We also obtained the GO terms significantly enriched in at least five conditions and KEGG pathways significantly enriched in at least three conditions (Figure S8C,D, Supporting Information), such as a component of mitochondrial membrane, RNA processing, and apoptosis. Especially, mitochondrial calcium ion regulation‐related functions were discovered in stomach adenocarcinoma conditions, which is associated with drug resistance to chemotherapy.^[^ ^46^ ^]^

**Figure 6:**
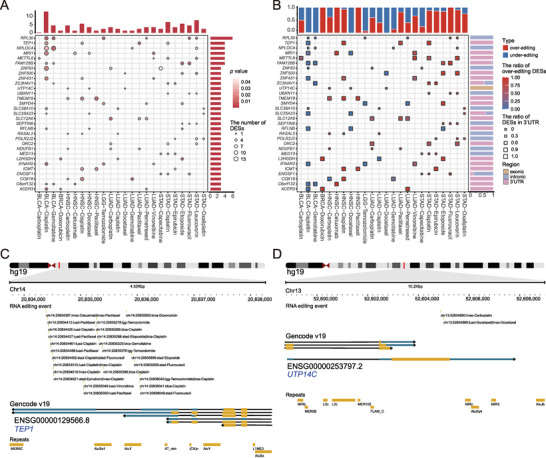
Genes enriched with differential editing sites (DESs). A) Genes significantly enriched with DESs under at least three conditions are shown. The color of the circles represents the statistical significance (*p*‐value). The size of the circles represents the number of DESs. The top barplot represents the number of genes significantly enriched with DESs under each condition, and the right barplot represents the number of conditions in which genes enriched with DESs exist. B) The ratio of over‐editing sites and transcribed region distribution of DESs on each gene under each condition. The color of the checks represents the proportion of over‐editing sites. The size of the checks represents the proportion of DESs in the 3’‐UTR. The top barplot represents the proportion of over‐editing sites on all enriched genes under each condition. The right barplot represents transcribed region distribution of DESs on one gene across all conditions. C) *TEP1* contains 34 unique DESs in the 3’‐UTR across 16 conditions. Here, only some DESs are displayed because of the relatively large number. D) *UTP14C* contains only two unique DESs in coding regions across three conditions.

To explore the editing patterns of genes enriched with DESs, we showed an editing overview of genes significantly enriched with DESs under at least three conditions (**Figure** **6**A,B). Notably, we found that the editing pattern and transcribed regions harboring DESs were unitary on a particular gene in each condition. For example, *TEP1* (telomerase associated protein 1, a component protein of human telomerase) has an under‐editing pattern in BLCA‐Cisplatin, where DESs were only located in the 3’‐UTR. Furthermore, the DESs on each enriched gene preferred to be in the same transcribed region across different conditions, especially in the 3’‐UTR and intronic regions (right barplot in Figure 6B). For example, *TEP1* harbored 34 unique DESs within its 3’‐UTR across 16 conditions (Figure 6C), which correlates with tumor cell sensitivity to Temozolomide.^[^ ^35^ ^]^ In contrast, there were two unique DESs in the exonic region of *UTP14C* across three conditions (Figure 6D), and *UTP14C* has been confirmed to protect tumor cells from chemotherapeutic drug‐induced apoptosis.^[^ ^36^ ^]^ In addition, the editing patterns of each enriched gene in resistant samples varied across different types of cancer, whereas RNA editing patterns of different genes were similar under the same condition (top barplot in Figure 6B). These findings implied that the mechanisms of drug resistance might be diverse in different types of cancer because of different editing patterns. Collectively, these results indicated that the alteration of RNA editing in specific transcribed regions of particular genes may impact the drug response in different conditions.

## 3 Discussion

Previous studies have reported that RNA editing could cause intra‐ and inter‐individual differences in drug response by affecting pharmacokinetics/pharmacodynamics‐related genes. More recently, the functional role of RNA editing in drug response has started to become clear. However, these studies were generally limited by the number of examined RNA editing sites, drugs, or tissues, which do not systematically analyze the RNA editing‐mediated mechanisms in drug response. Our study systematically investigated the landscape of A‐to‐I RNA editing associated with drug resistance across diverse conditions. Moreover, we revealed molecular regulatory mechanisms of drug resistance from the perspective of RNA editing, especially as a possible post‐transcriptional mechanism regulating the expression of critical genes.

In this study, we discovered that there is no significant difference in the OELs between resistant and sensitive samples (Figure 1D,E), this was also observed in cancer cell lines (Figure S2E,F, Supporting Information). However, we identified diversity of altered RNA editing events in resistant samples relative to sensitive samples across conditions (Figure 2A). We also characterized the DESs in resistant samples relative to sensitive samples. The DESs preferentially enriched in the 3’‐UTR (Figure 2B–D), whereas the DESs in the coding region were consistently limited (Figure 2E) across the conditions. Similar results were observed in cell lines (Figure S3, Supporting Information). These results parallel the recent finding that alteration of RNA editing in the 3’‐UTR of a particular gene could impact drug response. Previous studies have shown that patients with therapy resistance generally had poor outcomes. Therefore, we performed univariate Cox proportional hazards regression analysis to further validate the reliability of these editing sites associated with drug response. We observed that 859 of 7157 DESs were significantly associated with prognosis of the patients (Table S7, Supporting Information). Notably, editing patterns of 711 of 859 DESs were consistent between patients with poor prognosis and drug resistance (Table S7, Supporting Information). These findings suggested that RNA editing might be a crucial mediator of anticancer drug response. In addition, some of these DESs were also detected between resistant and sensitive cell lines (Table S3, Supporting Information). In particular, a large proportion of genes harboring DESs were simultaneously observed in tumor tissues and cell lines (Table S4, Supporting Information). Here, we discovered prominent diversity of the over‐ and under‐editing proportion of DESs under each condition (Figure 4A–C). These observations could only be partially explained by ADAR enzyme expression (Figure 4D–F). Furthermore, previous studies suggest that RBPs act as important mediators in regulating RNA editing. Here, we discovered that these DESs share common sequence motifs and overlap with binding sites of RBPs crucial for drug resistance (Figure 4G and Figure S5, Supporting Information). These findings indicated that RBPs are highly likely to play a potential role in the mechanism of drug resistance by regulating RNA editing. In addition, we customized a relatively rigid scheme to analyze the mechanisms by which DESs impact drug response by mediating miRNA‐gene relationships (Figure 5A). Therefore, our results required further studies regarding the RNA editing in miRNA modulations affecting drug response. For example, we revealed that over‐expression of *TRAF6* was possibly because of the lack of miR‐484_*TRAF6* interactions that may be due to the observed over‐editing in the 3’‐UTR of *TRAF6* in LGG‐Temozolomide resistant samples (Figure 5B–E).

Subsequently, we observed some drug resistance‐related GO terms and pathways, such as apoptosis, drug metabolism, Wnt signaling pathway, chemokine signaling pathway, DNA synthesis involved in DNA repair, etc. (Tables S12 and S13, Supporting Information), using function enrichment analysis of genes significantly enriched with DESs. Additionally, the functions of genes enriched with these DESs were specific under each condition (Figure S8A,B, Supporting Information), which implied that there are possibly different mechanisms of drug resistance between different conditions. Notably, the DESs on each enriched gene preferred to be in the same transcribed region across different conditions, especially in the 3’‐UTR and intronic regions (Figure 6B). Moreover, we discovered that the editing patterns of each enriched gene in resistant samples varied in different types of cancer. In contrast, RNA editing patterns of different genes were similar under the same condition (Figure 6B).

Currently, several studies confirmed RNA editing events related to drug resistance using experiments, consistent with our results. Here, we observed that the DESs on *DHFR* were located in the 3’‐UTR (Table S2, Supporting Information). A previous study has demonstrated that dysregulation of RNA editing sites in the 3’‐UTR of *DHFR* influences the binding of miR‐25‐3p and miR‐125a‐3p, enhancing cellular proliferation and resistance to methotrexate in MCF‐7 cells.^[^ ^21^ ^]^ We also found that the DESs on *COG3* are located in the coding region. Liang et al. confirmed that RNA editing in the coding region of *COG3* alters drug sensitivity using a high‐throughput Ba/F3 differential cytotoxicity screen.^[^ ^20^ ^]^ These evidence directly support our results. In future, the support of molecular biology experiments would improve the reliability of our results.

The RNA editing events in the exon region could change amino acid. However, there were only 19 unique nonsynonymous DESs identified in the current study. Most (76.5%) of the genes harboring these sites have been reported to be related to cancer progression and drug resistance (Table S6, Supporting Information). Previous studies confirmed that mutations could perturb the network, either by directly altering the normal functions of proteins (“nodetic” effect) or by altering the protein‐protein interactions (PPIs) (“edgetic” effect).^[^ ^47^ ^]^ Thus, we further explored whether these sites would affect PPIs. We mapped all 19 nonsynonymous DESs on PPI interface regions predicted by ECLAIR,^[^ ^48^ ^]^ and found that all nonsynonymous DESs load in PPI noninterface regions rather than interface regions. This may be due to few nonsynonymous DESs in the coding region. In future, we will specifically study the effect of editing sites on PPIs. At present, the protein structure and PPI landscape of personal cancer mutanomes has been illustrated,^[^ ^49^ ^]^ providing expert opinions on rational strategies for drug discovery and drug resistance for each tumor. Similar to DNA mutations, a personal RNA editome can also serve as a new source for the emerging development of personalized cancer medicine. In addition, recent studies demonstrated that the editing quantitative trait loci (edQTL) signatures co‐localize with complex disease genome‐wide association studies (GWAS) associations.^[^ ^24^ ^]^ However, there is a paucity of information on the co‐location of cancer GWAS loci and edQTL signatures. Previous studies have reported the pivotal effects of RNA editing in cancer progression, and several studies regarding cancer GWAS loci were reported. Therefore, it is necessary to explore edQTL signatures co‐localized with cancer loci in specific types of cancer in future studies.

However, there are several limitations of current RNA editing data and drug response data in vivo, particularly the number of examined RNA editing sites, drugs, samples, or tissues. We found that there was a positive correlation between the sample size and the number of identified DESs, suggesting that the sample size may affect the number of identified DESs. However, the current sample size with drug response information was relatively small. More accurate and comprehensive DESs can be obtained with the continuous increment of sample size in future. Recently, Yuan et al. developed a human endonuclease V mediated sequencing method for the single‐base resolution detection of A‐to‐I editing sites, which reduces false positives due to somatic mutations and single nucleotide polymorphisms.^[^ ^50^ ^]^ With the emergence of single‐base resolution methods and the accumulation of data, transcriptome‐wide A‐to‐I RNA editing information will be captured more accurately, which will provide more effective support for the functional exploration of A‐to‐I RNA editing. Further studies should be performed to improve specific RNA targeting for drug resistance‐associated RNA editing events in designing new RNA drugs. In summary, we systematically examined drug resistance‐related RNA editing events. We analyzed the mechanism of anticancer drug resistance from the perspective of RNA editing, which may improve the understanding of drug resistance mechanism regarding transcriptional and post‐transcriptional regulation.

## 4 Experimental Section

### Drug Response Data and RNA Editing Data of Samples

RNA editing data were downloaded from Synapse (syn2374375, https://www.synapse.org/), which was directly retrieved from RNA‐seq data of tumor samples in TCGA through a customized computational pipeline.^[^ ^13^, ^20^ ^]^ Here, the informative RNA editing sites identified in the previous study were used and further analyzed in this study.^[^ ^20^ ^]^ These informative RNA editing sites derived from tumor samples were re‐annotated using ANNOVAR (version‐Date: 2019‐10‐24). Drug response data of tumor samples in TCGA were obtained from the GDC Data Portal (https://portal.gdc.cancer.gov/). Thereafter, only samples with RNA editing profiles and drug responses were retained. Samples labeled “Progressive Disease” and “Complete Response” were defined as resistant and sensitive samples, respectively. One drug in a specific type of cancer was defined as a condition. Each condition included at least three tumor samples in the resistant and sensitive groups.

### Identification of Adenosine‐to‐Inosine RNA Editing Sites from RNA‐Sequencing Data of Cancer Cell Lines

Here, It was examined whether the DESs related to drug response in tumor samples were consistent with those in the cancer cell lines. The concentration at which growth was inhibited by 50% (IC50, half maximal inhibitory concentration) was generally used to measure the drug responses of many cancer cell lines.^[^ ^51^ ^]^ In this study, first, drug response data of cancer cell lines were downloaded from GDSC. *Z*‐score normalization for the ln(IC50) values was applied across the cell lines used for each compound. Thereafter, a cell line was defined as resistant or sensitive to the compound if the calculated *Z*‐score was >0.8 or <‐0.8, respectively. Cell lines with *Z*‐score between −0.8 and 0.8 were considered to be intermediate and were not used in further analyses. In addition, there were at least three resistant and three sensitive cell lines for each compound assayed under each condition, and the conditions in the cell lines had to be consistent with the analysis criterion in the samples. Finally, 14 conditions across five types of cancer and 11 compounds met this criterion, which involved 102 cell lines.

Thereafter, RNA‐seq raw data of 102 cell lines were downloaded from the CCLE (BioProject accession: PRJNA523380). The quality of the sequences using FastQC software (v0.11.9) was checked. Paired‐end reads were trimmed from the 3’ end to remove low‐quality bases using fastp (v0.20.1). Trimmed paired‐end reads were mapped on the human reference genome (hg19) using the STAR (v2.7.1a) program. Multi‐hits and duplicates were excluded from the read alignment. Aligned BAM files were used (REDItoolDenovo.py) to identify the editing level of RNA editing sites using the REDItools (v2) software with default parameters. Finally, editing profiles were retrieved by filtering out the RNA editing sites with multiple mismatches in each cell line.

### Overall RNA Editing Level Analysis

Here, the average editing levels in all informative RNA editing sites of each sample as the OEL of the sample were calculated. *R* ^2^ values and statistical significance (*p*‐values) were calculated using linear regressions to assess the correlations between OELs and ADAR expression. The differential analysis of OELs was based on the Wilcoxon rank‐sum test.

### Differential RNA Editing Analysis between Resistant and Sensitive Tumor Samples

In this study, informative RNA editing sites with no missing values in at least three resistant and three sensitive samples were required. Thereafter, the Wilcoxon rank‐sum test was used to detect RNA editing sites with differential editing activity between resistant and sensitive tumor samples. DESs as statistically significant with *p* < 0.05 and |*Diff*| ≥ 5% are defined. Here, |*Diff*| represents the difference of the mean editing level between resistant and sensitive samples. The gene expression (fragments per kilobase of transcript per million fragments mapped, FPKM) data of patients were downloaded from the GDC Data Portal. The differential expression of ADAR enzymes between resistant and sensitive samples was detected using Wilcoxon rank‐sum test.

### Transcribed Region Distribution of Differential Editing Sites

The transcribed region distribution of the DESs was investigated in an integrative manner and separately under each condition. It was examined whether the DESs were significantly enriched in the particular transcribed regions using the hypergeometric test in an integrative manner and separately for each condition. Furthermore, only the transcribed region with *p* < 0.05 was considered as a significant region enriched with DESs.

### Comparison of Differential Editing Sites across Different Conditions

To compare DESs among different conditions, the Simpson's index was calculated, that were separately based on DESs, genes harboring DESs, or GO terms enriched with these genes under each condition as follows:

(1) $$Index=\frac{{Cond}_{i}\cap {Cond}_{j}}{Min\left({Cond}_{i},{Cond}_{j}\right)}$$

where *Cond*_*i*_ and *Cond*_*j*_ represent the number of terms of a certain type (DESs, genes, or GO terms) under condition *i* and condition *j*, respectively.

### Prognosis Relevance of Differential Editing Sites

It was investigated whether the DESs associated with drug response could distinguish between patients with cancer with favorable or poor outcomes. First, six types of cancer samples with RNA editing levels from Synapse (syn2374375) were collected. The editing level of each site was converted to binary according to the median of editing levels in all samples. The clinical data of patients were downloaded from the GDC Data Portal. The univariate Cox proportional hazards regression model to identify the DESs that significantly (*p* < 0.05) correlated with patient prognosis was used. The log‐rank test to evaluate the statistically significant difference of prognoses between over‐editing and under‐editing groups was also used. The Kaplan–Meier survival curve from the R (v3.6.3) package survival (v3.2‐11) was used to plot the two groups.

### Identification of RNA‐Binding Proteins that Potentially Regulate RNA Editing Level of Differential Editing Sites

Recent studies have demonstrated that RBPs can regulate editing by binding specific sequence motifs around the RNA editing sites.^[^ ^31^ ^]^ Here, sequences of ± 20 bp relative to each DES were extracted under each condition to discover potential motifs that possibly interact with RNA editing enzyme complexes. These sequences were subsequently subjected to the MEME tool (v5.4.1, http://meme‐suite.org/) to identify motifs that were notably enriched within these sequences, regardless of their relative location on editing sites.^[^ ^52^ ^]^ MEME was run using the classic mode and limiting the search to only the top three motifs. The enrichment was measured relative to a random model based on the frequencies of letters in submitted sequences. In addition, FIMO_(v5.4.1) was used to scan a set of sequences for individual matches to the given motifs.^[^ ^29^ ^]^ Here, Alu was selected as input sequences and the top motif obtained was used as the input motif to detect whether there were significantly matching motifs in Alu repeat elements.

Thereafter, the common sequence motif was used to map binding sites of human RBPs using the RBPmap database (http://rbpmap.technion.ac.il/).^[^ ^53^ ^]^ The algorithm for mapping motifs on RNA sequences was based on the weighted‐rank approach, which considers the clustering propensity of binding sites and the overall tendency of regulatory regions to be conserved. In this study, a list of motifs obtained in each condition was independently submitted to the RBPmap database to detect motifs enriched in RBP targets.

### Differential Gene Expression Analysis

For the comparison between genes harboring DESs and DEGs in resistant tumor tissues relative to sensitive tumor tissues, differential gene analysis between resistant and sensitive samples was performed. First, the gene count data of patients from the GDC Data Portal were downloaded. Thereafter, DEGs were identified between resistant and sensitive tumor tissues using edgeR (v3.26.8) from R (v3.6.3) packages.^[^ ^54^ ^]^ The edgeR package was a Bioconductor package for examining the differential expression of count data. Finally, significant DEGs were defined as *p* < 0.05.

### Identification of Drug Response‐Related Differential Editing Site‐MicroRNA‐Gene Triplets

An integrative analysis to identify potential triples that include DESs, miRNAs, and genes (Figure 5A) was performed. The multistep analysis was performed as follows: 1) An edited site can lead to a gain or loss regulation between miRNA and target genes. Here, the miRNA‐gene interactions that existed in edited sequences, and not in corresponding unedited sequences, were defined as gain‐regulations. In contrast, miRNA‐gene interactions that existed in unedited sequences, and not in edited sequences, were defined as loss‐regulations. Thus, the gain or loss of miRNA regulations on the gene due to the editing of DESs in the 3’‐UTR of the genes using TargetScan (v7.0, Perl script targetscan_70.pl) were predicted.^[^ ^55^ ^]^ 2) If gain‐regulations attributed to the over‐editing of DESs occurred in resistant samples, it would be significantly negative correlation between the expression of miRNA and gene in resistant samples rather than sensitive samples. In contrast, if the loss‐regulations attributed to the over‐editing of DESs occurred in resistant samples, the expression of miRNA and gene would be significantly negatively correlated in sensitive samples rather than resistant samples. Hence, the correlation between expression of miRNA and gene within the identified gain‐ or loss‐regulations above in resistant and sensitive samples, respectively (Pearson correlation, *p* < 0.05) was calculated. 3) The expression of the gene was lower in resistant samples when the miRNA‐gene relationship was gained in resistant samples. In contrast, the expression of the gene was higher in resistant samples when this miRNA‐gene relationship was lost in resistant samples. Accordingly, the FC of the mean gene expression level between resistant and sensitive samples was computed under each condition. In particular, differential expression analysis of miRNAs between resistant and sensitive samples using the Wilcoxon rank‐sum test was performed. Furthermore, non‐significant differentially expressed miRNAs were only focused on to neglect their impacts on gene expression. Finally, these A‐to‐I RNA editing‐dependent miRNA regulation triplets that met the above three screening criteria were retained.

### Identification of Genes Enriched with Differential Editing Sites

In this study, genes significantly enriched with DESs beyond what could be expected by chance were identified. In brief, a hypergeometric test was used to examine the over‐representation of DESs within a particular gene. Furthermore, only the gene with *p* < 0.05 was considered a statistically significant gene enriched with DESs. The visualization of the DESs in a particular gene was shown in Figure 6C,D using JBrowse.^[^ ^56^ ^]^

### Gene Set Enrichment Analyses

The functional annotation and enrichment of all identified RBPs were performed using Metascape.^[^ ^57^ ^]^ In Metascape, the enrichment of KEGG pathways and GO terms relevant to cellular components, molecular functions, and biological processes using a one‐tailed hypergeometric distribution with Bonferroni's correction were assessed. In addition, all genes that were significantly enriched with DESs under each condition were subjected to functional annotation and enrichment using the R (v3.6.3) package clusterProfiler (v3.12.0).^[^ ^58^ ^]^ The clusterProfiler package provided two functions, enrichGO and enrichKEGG, to perform enrichment analysis for GO terms and KEGG pathways based on the hypergeometric test. Here, significantly differential GO terms and KEGG pathways with *p* < 0.05 under each condition were defined.

### Statistical Analysis

The RNA editing of cancer cell lines was identified using the REDItools (v2) with default parameters. All statistical analyses were performed using R (v3.6.3). For comparison between two groups, a Wilcoxon rank‐sum test was performed. A univariate Cox proportional hazards regression model was used to identify the DESs that significantly correlated with the prognosis of patient. The statistically significant difference of prognoses between over‐editing and under‐editing groups using the log‐rank test was also evaluated. Genes significantly enriched with DESs beyond what could be expected by chance using the hypergeometric test were identified. A *p*‐value less than 0.05 established statistical significance.

## Conflict of Interest

The authors declare no conflict of interest.

## Author Contributions

W.J. and L.W. conceived and designed the study; X.Z. performed the data analysis and website construction and drafted the manuscript; R.M., F.H., and S.Z. discussed the results and revised the manuscript. All authors reviewed, revised, and approved the manuscript.

## Supporting information

Supporting Information

Supplemental Table 1

Supplemental Table 2

Supplemental Table 3

Supplemental Table 4

Supplemental Table 5

Supplemental Table 6

Supplemental Table 7

Supplemental Table 8

Supplemental Table 9

Supplemental Table 10

Supplemental Table 11

Supplemental Table 12

Supplemental Table 13

## Data Availability

The data that support the findings of this study are available in the supplementary material of this article.

## References

[1] a) H. Furue , Cancer Chemother. 2003, 30, 1404; 14584272

[2] a) N. Vasan , J. Baselga , D. M. Hyman , Nature 2019, 575, 299; 31723286 10.1038/s41586-019-1730-1 PMC8008476

[3] S. M. Jeon , E. A. Shin , Exp. Mol. Med. 2018, 50, 1. 10.1038/s12276-018-0038-9 PMC5938036 29657326

[4] S. Yao , L. Y. Fan , E. W. Lam , Semin. Cancer Biol. 2018, 50, 77. 29180117 10.1016/j.semcancer.2017.11.018 PMC6565931

[5] C. Gourley , J. Balmana , J. A. Ledermann , V. Serra , R. Dent , S. Loibl , E. Pujade‐Lauraine , S. J. Boulton , J. Clin. Oncol. 2019, 37, 2257. 31050911 10.1200/JCO.18.02050

[6] T. Wu , Y. Dai , Cancer Lett. 2017, 387, 61. 26845449 10.1016/j.canlet.2016.01.043

[7] L. Wei , J. Sun , N. Zhang , Y. Zheng , X. Wang , L. Lv , J. Liu , Y. Xu , Y. Shen , M. Yang , Mol. Cancer 2020, 19, 62. 32192494 10.1186/s12943-020-01185-7 PMC7081551

[8] C. Holohan , S. Van Schaeybroeck , D. B. Longley , P. G. Johnston , Nat. Rev. Cancer 2013, 13, 714. 24060863 10.1038/nrc3599

[9] L. Garcia‐Martinez , Y. Zhang , Y. Nakata , H. L. Chan , L. Morey , Nat. Commun. 2021, 12, 1786. 33741974 10.1038/s41467-021-22024-3 PMC7979820

[10] Q. Lan , P. Y. Liu , J. L. Bell , J. Y. Wang , S. Huttelmaier , X. D. Zhang , L. Zhang , T. Liu , Cancer Res. 2021, 81, 3431. 34228629 10.1158/0008-5472.CAN-20-4107

[11] C. R. Walkley , J. B. Li , Genome Biol. 2017, 18, 205. 29084589 10.1186/s13059-017-1347-3 PMC5663115

[12] a) E. Eisenberg , E. Y. Levanon , Nat. Rev. Genet. 2018, 19, 473; 29692414 10.1038/s41576-018-0006-1

[13] a) E. Picardi , A. M. D'Erchia , C. Lo Giudice , G. Pesole , Nucleic Acids Res. 2017, 45, D750; 27587585 10.1093/nar/gkw767 PMC5210607

[14] H. Liu , J. Golji , L. K. Brodeur , F. S. Chung , J. T. Chen , R. S. deBeaumont , C. P. Bullock , M. D. Jones , G. Kerr , L. Li , D. P. Rakiec , M. R. Schlabach , S. Sovath , J. D. Growney , R. A. Pagliarini , D. A. Ruddy , K. D. MacIsaac , J. M. Korn , E. R. McDonald III , Nat. Med. 2019, 25, 95. 30559422 10.1038/s41591-018-0302-5

[15] M. Kubota‐Sakashita , K. Iwamoto , M. Bundo , T. Kato , Mol. Brain 2014, 7, 5. 24443933 10.1186/1756-6606-7-5 PMC3902024

[16] K. Stellos , A. Gatsiou , K. Stamatelopoulos , L. Perisic Matic , D. John , F. F. Lunella , N. Jae , O. Rossbach , C. Amrhein , F. Sigala , R. A. Boon , B. Furtig , Y. Manavski , X. You , S. Uchida , T. Keller , J. N. Boeckel , A. Franco‐Cereceda , L. Maegdefessel , W. Chen , H. Schwalbe , A. Bindereif , P. Eriksson , U. Hedin , A. M. Zeiher , S. Dimmeler , Nat. Med. 2016, 22, 1140. 27595325 10.1038/nm.4172

[17] M. Jain , M. F. Jantsch , K. Licht , Trends Genet. 2019, 35, 903. 31648814 10.1016/j.tig.2019.09.004

[18] G. Niu , D. Zou , M. Li , Y. Zhang , J. Sang , L. Xia , M. Li , L. Liu , J. Cao , Y. Zhang , P. Wang , S. Hu , L. Hao , Z. Zhang , Nucleic Acids Res. 2019, 47, D78. 30357418 10.1093/nar/gky958 PMC6323952

[19] a) M. Nakano , M. Nakajima , Pharmacol. Ther. 2018, 181, 13; 28716651 10.1016/j.pharmthera.2017.07.003

[20] L. Han , L. Diao , S. Yu , X. Xu , J. Li , R. Zhang , Y. Yang , H. M. J. Werner , A. K. Eterovic , Y. Yuan , J. Li , N. Nair , R. Minelli , Y. H. Tsang , L. W. T. Cheung , K. J. Jeong , J. Roszik , Z. Ju , S. E. Woodman , Y. Lu , K. L. Scott , J. B. Li , G. B. Mills , H. Liang , Cancer Cell 2015, 28, 515. 26439496 10.1016/j.ccell.2015.08.013 PMC4605878

[21] M. Nakano , T. Fukami , S. Gotoh , M. Nakajima , J. Biol. Chem. 2017, 292, 4873. 28188287 10.1074/jbc.M117.775684 PMC5377802

[22] a) H. Song , D. Liu , S. Dong , L. Zeng , Z. Wu , P. Zhao , L. Zhang , Z. S. Chen , C. Zou , Signal Transduction Targeted Ther. 2020, 5, 193; 10.1038/s41392-020-00300-w PMC7479143 32900991

[23] M. H. Tan , Q. Li , R. Shanmugam , R. Piskol , J. Kohler , A. N. Young , K. I. Liu , R. Zhang , G. Ramaswami , K. Ariyoshi , A. Gupte , L. P. Keegan , C. X. George , A. Ramu , N. Huang , E. A. Pollina , D. S. Leeman , A. Rustighi , Y. P. S. Goh , G. T. Consortium , A. Chawla , G. Del Sal , G. Peltz , A. Brunet , D. F. Conrad , C. E. Samuel , M. A. O'Connell , C. R. Walkley , K. Nishikura , J. B. Li , Nature 2017, 550, 249.29022589

[24] M. S. Breen , A. Dobbyn , Q. Li , P. Roussos , G. E. Hoffman , E. Stahl , A. Chess , P. Sklar , J. B. Li , B. Devlin , J. D. Buxbaum , C. CommonMind , Nat. Neurosci. 2019, 22, 1402. 31455887 10.1038/s41593-019-0463-7 PMC6791127

[25] D. W. Shen , L. M. Pouliot , J. P. Gillet , W. Ma , A. C. Johnson , M. D. Hall , M. M. Gottesman , Mol. Pharmaceutics 2012, 9, 1822. 10.1021/mp300153z PMC3367311 22571463

[26] F. Jun , Z. Peng , Y. Zhang , D. Shi , Gynecol. Oncol. 2020, 157, 268. 31955862 10.1016/j.ygyno.2020.01.013

[27] X. Liu , G. Li , L. Ai , Q. Ye , T. Yu , B. Yang , Oncol. Lett. 2019, 18, 1304. 31423190 10.3892/ol.2019.10454 PMC6607384

[28] X. Jiang , Y. Xia , H. Meng , Y. Liu , J. Cui , H. Huang , G. Yin , B. Shi , Front. Oncol. 2021, 11, 746029. 34692528 10.3389/fonc.2021.746029 PMC8528313

[29] C. E. Grant , T. L. Bailey , W. S. Noble , Bioinformatics 2011, 27, 1017. 21330290 10.1093/bioinformatics/btr064 PMC3065696

[30] a) Y. Wang , A. J. Bernhardy , C. Cruz , J. J. Krais , J. Nacson , E. Nicolas , S. Peri , H. van der Gulden , I. van der Heijden , S. W. O'Brien , Y. Zhang , M. I. Harrell , S. F. Johnson , F. J. C. Dos Reis , P. D. Pharoah , B. Karlan , C. Gourley , D. Lambrechts , G. Chenevix‐Trench , H. Olsson , J. J. Benitez , M. H. Greene , M. Gore , R. Nussbaum , S. Sadetzki , S. A. Gayther , S. K. Kjaer , I. kConFab , A. D. D'Andrea , G. I. Shapiro , et al., Cancer Res. 2016, 76, 2778; 27197267 10.1158/0008-5472.CAN-16-0186 PMC4874568

[31] E. C. Freund , A. L. Sapiro , Q. Li , S. Linder , J. J. Moresco , J. R. Yates III , J. B. Li , Cell Rep. 2020, 31, 107656. 32433965 10.1016/j.celrep.2020.107656 PMC7306178

[32] G. Quinones‐Valdez , S. S. Tran , H. I. Jun , J. H. Bahn , E. W. Yang , L. Zhan , A. Brummer , X. Wei , E. L. Van Nostrand , G. A. Pratt , G. W. Yeo , B. R. Graveley , X. Xiao , Commun. Biol. 2019, 2, 19. 30652130 10.1038/s42003-018-0271-8 PMC6331435

[33] C. Chen , M. Zhang , Y. Zhang , Cell Transplant. 2020, 29, 096368972096107. 10.1177/0963689720961070 PMC7784611 32951448

[34] Z. Qian , S. Zhou , Z. Zhou , X. Yang , S. Que , J. Lan , Y. Qiu , Y. Lin , Oncol. Rep. 2017, 38, 2941. 29048680 10.3892/or.2017.5970

[35] T. Kanzawa , I. M. Germano , Y. Kondo , H. Ito , S. Kyo , S. Kondo , Br. J. Cancer 2003, 89, 922. 12942127 10.1038/sj.bjc.6601193 PMC2394478

[36] T. Ma , C. Lu , Y. Guo , C. Zhang , X. Du , Biol. Chem. 2017, 398, 1247. 28672776 10.1515/hsz-2017-0121

[37] S. B. Koh , K. Ross , S. J. Isakoff , N. Melkonjan , L. He , K. J. Matissek , A. Schultz , E. L. Mayer , T. A. Traina , L. A. Carey , H. S. Rugo , M. C. Liu , V. Stearns , A. Langenbucher , S. V. Saladi , S. Ramaswamy , M. S. Lawrence , L. W. Ellisen , Clin. Cancer. Res. 2021, 27, 4883. 34168046 10.1158/1078-0432.CCR-21-0714 PMC8416935

[38] A. Tomida , T. Tsuruo , Anticancer Drug Des. 1999, 14, 169. 10405643

[39] B. D. Wang , N. H. Lee , Cancers 2018, 10, 458.30463359

[40] a) B. A. Carneiro , W. S. El‐Deiry , Nat. Rev. Clin. Oncol. 2020, 17, 395; 32203277 10.1038/s41571-020-0341-y PMC8211386

[41] a) H. Verma , M. S. Bahia , S. Choudhary , P. K. Singh , O. Silakari , Drug Metab. Rev. 2019, 51, 196; 31203662 10.1080/03602532.2019.1632886

[42] Z. Zhong , D. M. Virshup , Mol. Pharmacol. 2020, 97, 72. 31787618 10.1124/mol.119.117978

[43] M. E. Reyes , M. de La Fuente , M. Hermoso , C. G. Ili , P. Brebi , Front. Immunol. 2020, 11, 901. 32499779 10.3389/fimmu.2020.00901 PMC7243460

[44] a) L. E. Wang , M. Yin , Q. Dong , D. J. Stewart , K. W. Merriman , C. I. Amos , M. R. Spitz , Q. Wei , J. Clin. Oncol. 2011, 29, 4121; 21947825 10.1200/JCO.2010.34.3616 PMC3675702

[45] D. Delbue , B. S. Mendonca , M. C. Robaina , L. G. T. Lemos , P. I. Lucena , J. P. B. Viola , L. M. Magalhaes , S. Crocamo , C. A. B. Oliveira , F. R. Teixeira , R. C. Maia , G. N. de Moraes , Biochim. Biophys. Acta, Mol. Cell Res. 2020, 1867, 118761. 32485270 10.1016/j.bbamcr.2020.118761

[46] V. Tangeda , Y. K. Lo , A. P. Babuharisankar , H. Y. Chou , C. L. Kuo , Y. H. Kao , A. Y. Lee , J. Y. Chang , Cell Death Dis. 2022, 13, 241. 35296653 10.1038/s41419-022-04668-1 PMC8927349

[47] a) F. Cheng , J. Zhao , Y. Wang , W. Lu , Z. Liu , Y. Zhou , W. R. Martin , R. Wang , J. Huang , T. Hao , H. Yue , J. Ma , Y. Hou , J. A. Castrillon , J. Fang , J. D. Lathia , R. A. Keri , F. C. Lightstone , E. M. Antman , R. Rabadan , D. E. Hill , C. Eng , M. Vidal , J. Loscalzo , Nat. Genet. 2021, 53, 342; 33558758 10.1038/s41588-020-00774-y PMC8237108

[48] M. J. Meyer , J. F. Beltran , S. Liang , R. Fragoza , A. Rumack , J. Liang , X. Wei , H. Yu , Nat. Methods 2018, 15, 107. 29355848 10.1038/nmeth.4540 PMC6026581

[49] a) R. Nussinov , H. Jang , G. Nir , C. J. Tsai , F. Cheng , Signal Transduction Targeted Ther. 2021, 6, 3; 10.1038/s41392-020-00420-3 PMC7785737 33402669

[50] J. J. Chen , X. J. You , L. Li , N. B. Xie , J. H. Ding , B. F. Yuan , Y. Q. Feng , Anal. Chem. 2022, 94, 8740. 35678728 10.1021/acs.analchem.2c01226

[51] a) F. Iorio , T. A. Knijnenburg , D. J. Vis , G. R. Bignell , M. P. Menden , M. Schubert , N. Aben , E. Goncalves , S. Barthorpe , H. Lightfoot , T. Cokelaer , P. Greninger , E. van Dyk , H. Chang , H. de Silva , H. Heyn , X. Deng , R. K. Egan , Q. Liu , T. Mironenko , X. Mitropoulos , L. Richardson , J. Wang , T. Zhang , S. Moran , S. Sayols , M. Soleimani , D. Tamborero , N. Lopez‐Bigas , P. Ross‐Macdonald , et al., Cell 2016, 166, 740; 27397505 10.1016/j.cell.2016.06.017 PMC4967469

[52] T. L. Bailey , C. Elkan , Proc. Int. Conf. Intell. Syst. Mol. Biol. 1994, 2, 28. 7584402

[53] I. Paz , I. Kosti , M. Ares Jr. , M. Cline , Y. Mandel‐Gutfreund , Nucleic Acids Res. 2014, 42, W361. 24829458 10.1093/nar/gku406 PMC4086114

[54] M. D. Robinson , D. J. McCarthy , G. K. Smyth , Bioinformatics 2010, 26, 139. 19910308 10.1093/bioinformatics/btp616 PMC2796818

[55] V. Agarwal , G. W. Bell , J. W. Nam , D. P. Bartel , Elife 2015, 4, e05005. 26267216 10.7554/eLife.05005 PMC4532895

[56] R. Buels , E. Yao , C. M. Diesh , R. D. Hayes , M. Munoz‐Torres , G. Helt , D. M. Goodstein , C. G. Elsik , S. E. Lewis , L. Stein , I. H. Holmes , Genome Biol. 2016, 17, 66. 27072794 10.1186/s13059-016-0924-1 PMC4830012

[57] Y. Zhou , B. Zhou , L. Pache , M. Chang , A. H. Khodabakhshi , O. Tanaseichuk , C. Benner , S. K. Chanda , Nat. Commun. 2019, 10, 1523. 30944313 10.1038/s41467-019-09234-6 PMC6447622

[58] G. Yu , L. G. Wang , Y. Han , Q. Y. He , OMICS 2012, 16, 284. 22455463 10.1089/omi.2011.0118 PMC3339379

