# Supplementary material for: Genomic Landscape and Potential Regulation of RNA Editing in Drug Resistance
Source: Adv Sci (Weinh). 2023 Mar 13;10(14):2207357. doi: 10.1002/advs.202207357 (PMC10190536; doi:10.1002/advs.202207357)
Supplement: Supplementary file 1 — Supporting Information [file ADVS-10-2207357-s002.pdf]

SUPPLEMENTAL FIGURES

Genomic Landscape and Potential Regulation of RNA Editing in Drug Resistance

Xu Zhou[1], Ramkrishna Mitra[2], Fei Hou[1], Shunheng Zhou[1], Lihong Wang[3*], Wei Jiang[1*]

[1] Department of Biomedical Engineering, Nanjing University of Aeronautics and Astronautics, Nanjing 211106, China
[2] Department of Pharmacology, Physiology, and Cancer Biology, Sidney Kimmel Cancer Center, Thomas Jefferson University, United States.
[3] Department of Pathophysiology, School of Medicine, Southeast University, Nanjing 210009, China
* Corresponding author
Correspondence should be addressed to W.J. (weijiang@nuaa.edu.cn) and L.W. (lw2247@yeah.net).

Supplemental Figures 1-8, in brief:

Figure S1. Number of the resistant and sensitive samples in each condition.

Figure S2. The OELs in resistant and sensitive cell lines.

Figure S3. Transcribed region distribution of DESs in cell lines.

Figure S4. Comparisons of DESs, genes harboring DESs and GO terms enriched with the genes between any two conditions.

Figure S5. The function of RBPs that potentially regulate the editing level of DESs.

Figure S6. The associations between site editing level and gene expression in each condition.

Figure S7. The comparison between genes harboring DESs and DEGs in resistant samples relative to sensitive samples in each condition.

Figure S8. The enriched functions and pathways of genes enriched with the DESs.

Supplemental Fig. S1

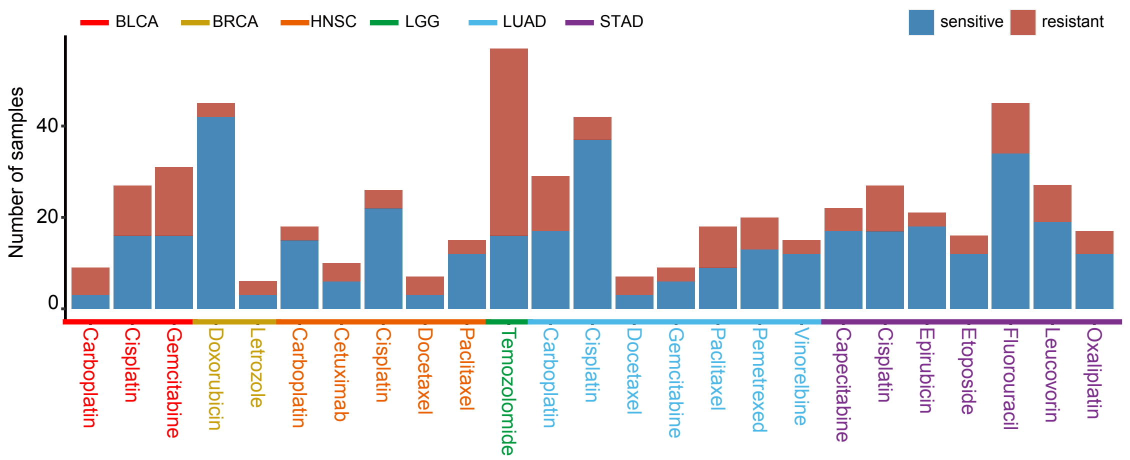

Figure S1. Number of the resistant and sensitive samples in each condition.

Supplemental Fig. S2

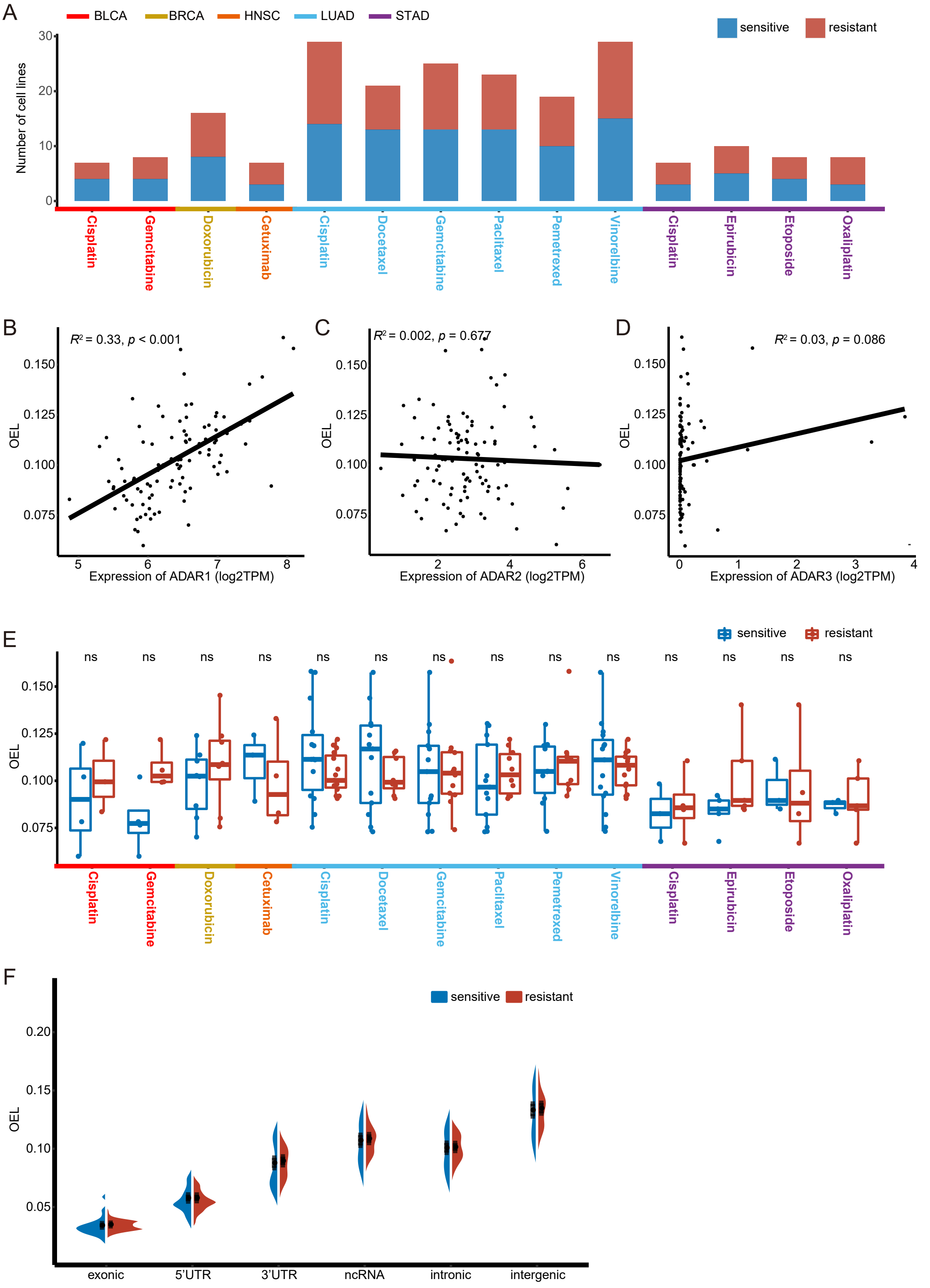

Figure S2. The OELs in resistant and sensitive cell lines. A, The number of resistant and sensitive cell lines used in this study. B-D, Correlations between expression levels (log2TPM) of ADAR1(B), ADAR2(C), and ADAR3(D) and OELs in cell lines were shown, respectively. E, The comparison of the OELs between compared groups was shown, respectively. F, The comparison of OELs in different transcribed regions between compared groups.

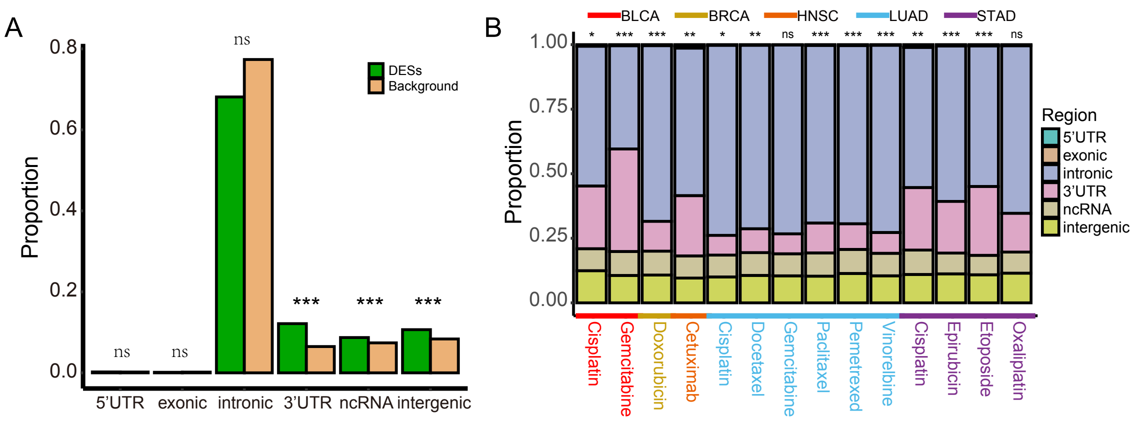

Figure S3. Transcribed region distribution of DESs in cell lines. A, DESs significantly enriched in 3'-UTR, ncRNA and intergenic regions in cell lines through the hypergeometric test. B, The transcribed region distribution of DESs in each condition from cell lines. The p-values were calculated through the hypergeometric test to measure whether DESs significantly enriched in 3'-UTR in each condition. Particularly, "*" represents $p < 0.05$, "**" represents $p < 0.01$, "***" represents $p < 0.001$, and "ns" represents "not significant".

Supplemental Fig. S4

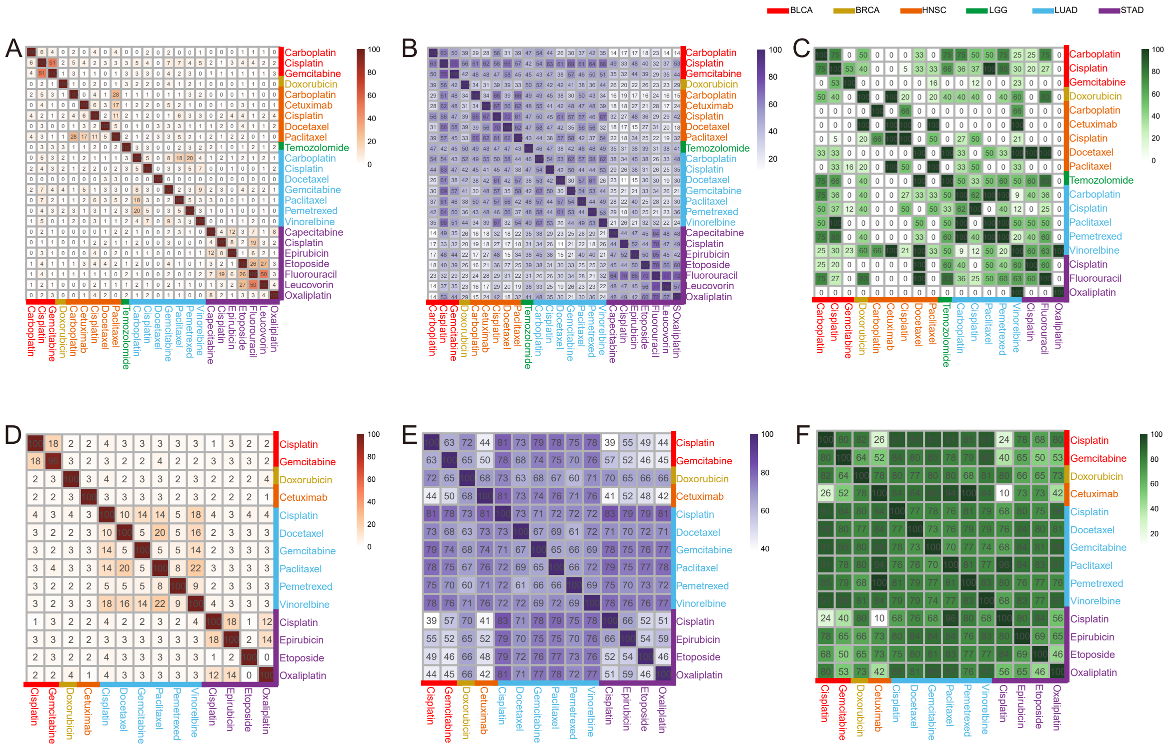

Figure S4. Comparisons of DESs, genes harboring DESs and GO terms enriched with the genes between any two conditions. The Simpson indexes were calculated between any two conditions. A-C, Comparisons between any two conditions from samples were based on DESs(A), genes harboring DESs(B), and GO terms(C) enriched with these genes. D-F, Comparisons between any two conditions from cell lines based on DESs(D), genes harboring DESs(E), and GO terms(F) enriched with these genes.

Supplemental Fig. S5

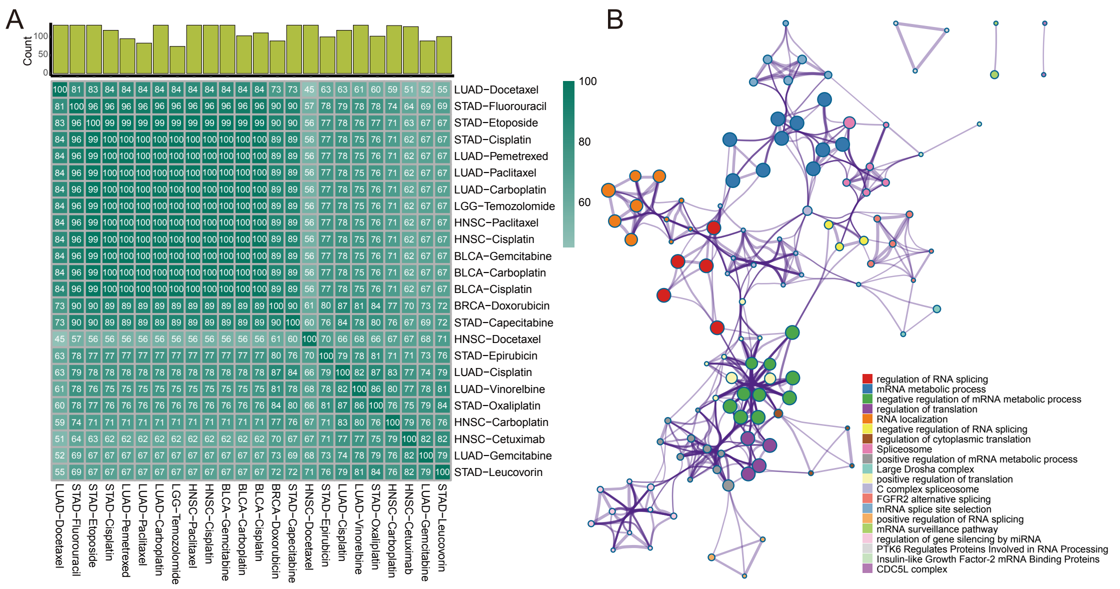

Figure S5. The function of RBPs that potentially regulate the editing level of DESs. A, Comparison of identified RBPs between any two conditions through Simpson index. B, The significantly enriched functions of all 132 RBPs through Metascape analysis.

Supplemental Fig. S6

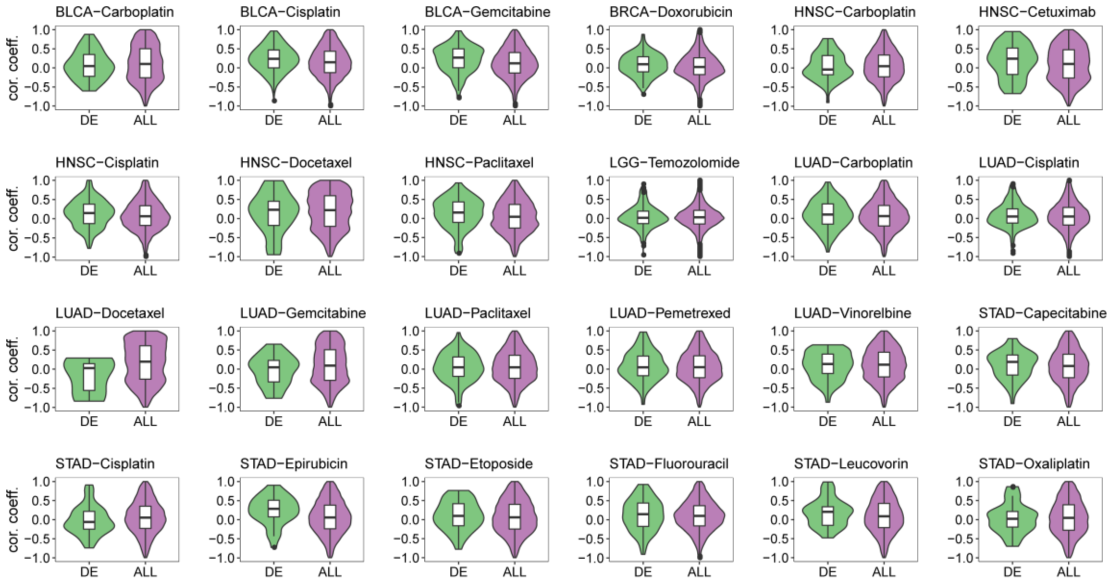

Figure S6. The associations between site editing level and gene expression in each condition. The Pearson correlation was calculated between editing level of each site and expression of the gene harboring this site. Particularly, ‘cor. coeff.’ represents ‘correlation coefficient’. Green represents Pearson correlation coefficients for DESs, and purple represents Pearson correlation coefficients for all informative editing sites.

Supplemental Fig. S7

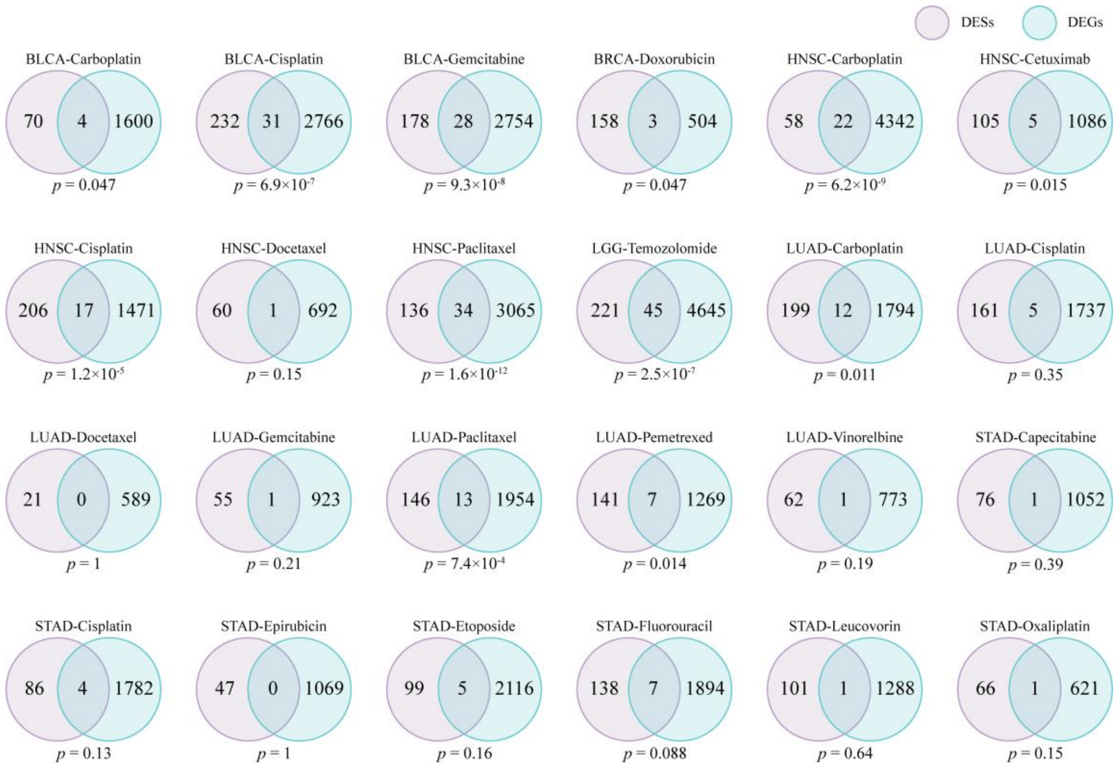

Figure S7. The comparison between genes harboring DESs and DEGs in resistant samples relative to sensitive samples in each condition. The *p*-values were calculated based on the hypergeometric test.

Supplemental Fig. S8

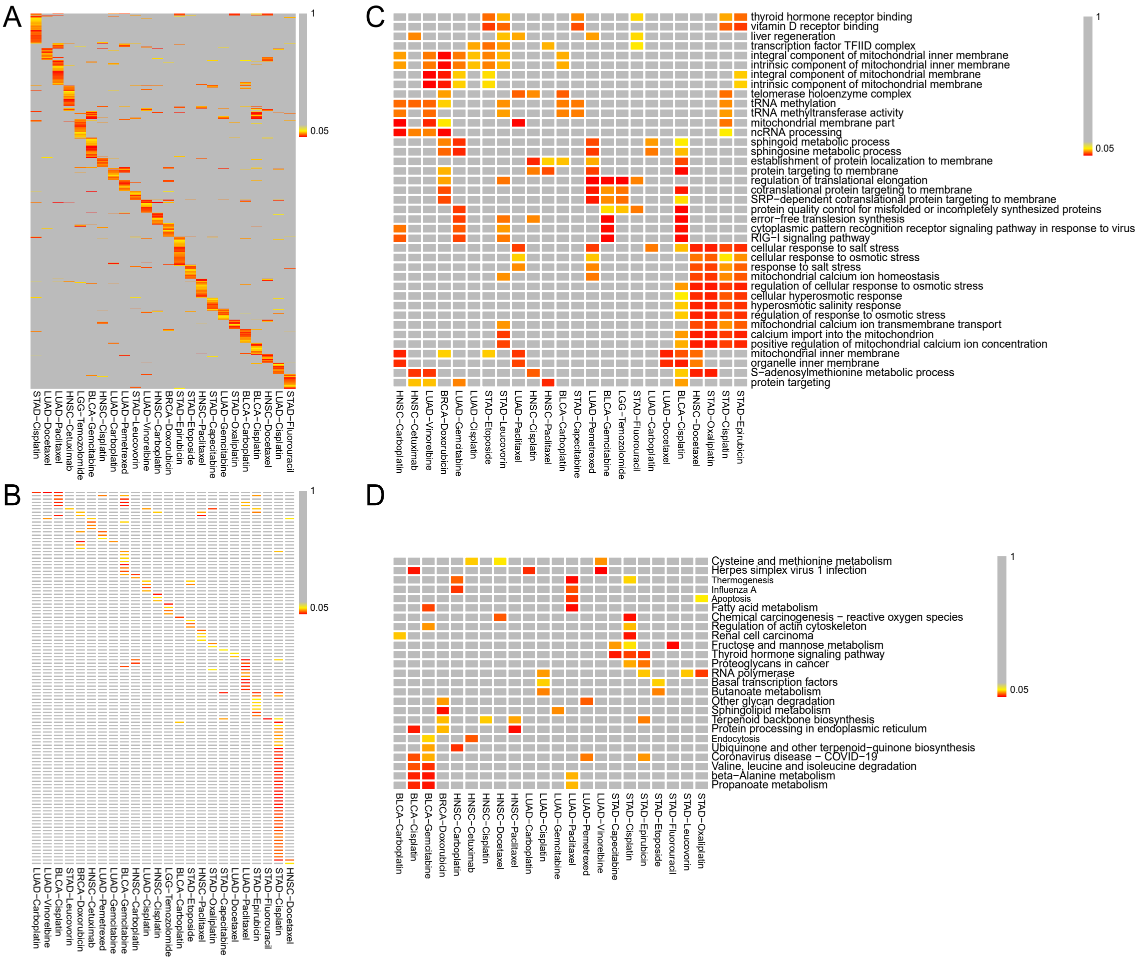

Figure S8. The enriched functions and pathways of genes enriched with the DESs. A, The significantly enriched GO terms in each condition ($p < 0.05$). B, The significantly enriched KEGG pathways in each condition ($p < 0.05$). C, The significant GO terms exist in at least five condition. D, The significant KEGG pathways exist in at least three conditions.
